# Anticancer activity of taraxerol acetate in human glioblastoma cells and a mouse xenograft model via induction of autophagy and apoptotic cell death, cell cycle arrest and inhibition of cell migration

**DOI:** 10.3892/mmr.2021.12100

**Published:** 2021-04-19

**Authors:** Jing-Fang Hong, Ying-Fang Song, Zheng Liu, Zhao-Cong Zheng, Hong-Jie Chen, Shou-Sen Wang

Mol Med Rep 13: 4541-4548, 2016; DOI: 10.3892/mmr.2016.5105

Following the publication of the above paper, a concerned reader drew to the Editor's attention that several figures (Figs. 3, 4, 7 and 10) contained apparent anomalies, including repeated patternings of data within the same figure panels. Furthermore, Fig. 3 contained data that bore striking similarities to data published in Fig. 6 in another paper published in *Molecular Medicine Reports*, which has now been retracted [Zhu Y-Y, Huang H-Y and Wu Y-L: Anticancer and apoptotic activities of oleanolic acid are mediated through cell cycle arrest and disruption of mitochondrial membrane potential in HepG2 human hepatocellular carcinoma cells. Mol Med Rep 12: 5012-5018, 2015].

After having conducted an independent investigation in the Editorial Office, the Editor of *Molecular Medicine Reports* has determined that the above paper should be retracted from the Journal on account of a lack of confidence concerning the originality and the authenticity of the data. The authors were asked for an explanation to account for these concerns, but the Editorial Office never received any reply. The Editor regrets any inconvenience that has been caused to the readership of the Journal.

